# Factors influencing secondary school students’ reading literacy: An analysis based on XGBoost and SHAP methods

**DOI:** 10.3389/fpsyg.2022.948612

**Published:** 2022-09-23

**Authors:** Hao Liu, Xi Chen, Xiaoxiao Liu

**Affiliations:** ^1^Collaborative Innovation Centre for Assessment of Basic Education Quality, Beijing Normal University, Beijing, China; ^2^Institute of Education, University of Alberta, Edmonton, AB, Canada

**Keywords:** reading literacy, Influencing factors, XGBoost, SHAP, interpretability\keywordbelowspace-30pt

## Abstract

This paper constructs a predictive model of student reading literacy based on data from students who participated in the Program for International Student Assessment (PISA 2018) from four provinces/municipalities of China, i.e., Beijing, Shanghai, Jiangsu and Zhejiang. We calculated the contribution of influencing factors in the model by using eXtreme Gradient Boosting (XGBoost) algorithm and sHapley additive exPlanations (SHAP) values, and get the following findings: (1) Factors that have the greatest impact on students’ reading literacy are from individual and family levels, with school-level factors taking a relative back seat. (2) The most important influencing factors at individual level are reading metacognition and reading interest. (3) The most important factors at family level are ESCS (index of economic, social and cultural status) and language environment, and dialect is negative for reading literacy, whereas proficiency in both a dialect and Mandarin plays a positive role. (4) At the school level, the most important factors are time dedicated to learning and class discipline, and we found that there is an optimal value for learning time, which suggests that reasonable learning time is beneficial, but overextended learning time may make academic performance worse instead of improving it.

## Introduction

Reading literacy is important for gaining knowledge and understanding the world, and it is a prerequisite for individual to become a good reader (Dreher and Mikulecky., 2000). The Program for International Student Assessment 2018 (PISA 2018) defined reading literacy as “understanding, using, evaluating, reflecting on and engaging with texts in order to achieve one’s goals, to develop one’s knowledge and potential and to participate in society.” PISA 2018 takes reading literacy as a foundation for full participation in contemporary society, requiring students to be able to integrate and put into practice textual information with prior knowledge while weighing the accuracy of arguments in and reflecting on the information conveyed by the text (OECD, 2019). As seen in the PISA definition, today’s reading literacy is no longer a skill acquired only in the early years of education but an evolving skill and strategy, and it’s focus is no longer on collection and memorization but on acquisition and use of information (OECD, 2010).

The results of the OECD Adult Skills Survey showed that literacy, numeracy and problem-solving skills are key information-processing skills for workers in the 21st century, and the survey found that workers who can make complex inferences and evaluate text claims and arguments are able to earn higher salaries than other workers, while workers with lower literacy skills face a higher risk of unemployment (OECD, 2013). This suggests that reading literacy has become a prerequisite for individuals to successfully participate in life and work. In addition to being important to workers themselves, reading literacy is also crucial for enhancing a nation’s cultural soft power and competitiveness and is an important indicator of a nation’s social civilization and comprehensive national power (Luo et al., 2016). Identifying the factors that significantly influence reading literacy and understanding factors influencing reading literacy would help students improve their reading literacy, which are crucial to the development of education policy making, top-level curriculum design, and improvement in classroom teaching strategies.

Students’ reading literacy is influenced by a variety of factors, including learning strategies, motivation, family support, school instruction, etc. These factors can be divided basically into three levels, i.e., individual level, family level, and school level.

### Factors at individual level

The impact of individual-level factors on reading literacy has been an important topic of research because its influence on student’s academic performance is direct. Existing research on students’ individual factors mostly centered on innate factors (e.g., intelligence, gender), reading strategies (e.g., learning strategies, metacognitive strategies), and motivation (e.g., interest in reading, competitive environment).

#### Innate factors

Deary et al. (2007) found a high correlation between intelligence traits and academic achievement for all subjects, including reading. Lechner et al. (2019) found that fluid intelligence positively predicted initial levels of reading ability and competence in later days. Smith et al. (2012) noted that girls’ reading achievement and enjoyment were significantly higher than that of boys’, implying a gender difference in reading literacy that cannot be ignored. By analyzing PISA 2018 data, Chunjin (2020), similarly, found significant gender differences in students’ reading literacy performance in four provinces/municipalities in China, but the effects were not as significant as other factors, while Logan (2008) noted small gender differences in reading ability but large gender differences in attention to and attitudes toward reading, and reading frequency. It has been claimed that gender affects students’ academic self-concept, motivation, and cognitive strategies (Swalander and Taube, 2007). Although both reading literacy and other factors showed significant gender differences, it has been suggested that stereotypes are one of source of gender differences due to the presence of stereotypes, as teachers usually have greater academic expectations of girls (Muntoni and Retelsdorf, 2018), which undermine boys’ self-concept of reading (Retelsdorf et al., 2015).

#### Reading strategies

Ülle et al. (2015) argued that learning strategies are very closely related to reading level and that students’ use of learning strategies can effectively explain the differences on reading literacy tests. Ghafournia and Afghari (2013) found that students at a high level of reading proficiency would use learning strategies more efficiently and stated that learning strategies can act as mediators to link linguistic and nonlinguistic variables, i.e., nonlinguistic variables indirectly affect linguistic variables through learning strategies. Reading metacognitive strategy measured the level of students’ perceptions of effective reading strategies (Jing, 2012), and the ability to use metacognitive strategies is closely related to students’ literacy performance (Paris and Oka, 1986; Lee and Shute, 2010; Areepattamannil and Caleon, 2013). In an experimental study, Paris and Oka (1986) found that increasing students’ metacognitive knowledge of reading can increase their use of reading strategies. Similarly, Jing (2012) analyzed data from Shanghai PISA 2009 and concluded that metacognitive strategies had a highly significant effect on Shanghai students’ reading performance.

#### Reading motivation

Fengning et al. (2000) found that there is a highly significant positive correlation between the level of secondary school students’ reading motivation and reading performance. Retelsdorf et al. (2011) divided reading motivation into intrinsic (e.g., reading enjoyment, reading interest) and extrinsic (e.g., competition) and found that reading enjoyment had a positive effect on initial reading performance but did not affect its further improvement, whereas interest in reading was not related to initial reading level but significantly affected the improvement of reading level, whereas competition has a negative effect on reading performance but does not affect its improvement, which is consistent with the findings in the research of Unrau and Schlackman (2006). Logan et al. (2011) argued that intrinsic motivation explains differences in the improvement of reading skills among students with low reading proficiency. And using quantile regression, Chunjin (2020), similarly, found that interest in reading has a greater marginal effect on reading literacy for students at the lower quantile. In addition, Pokay and Blumenfeld (1990) found that the pattern of motivation that affects academic performance by influencing learning strategies differed between early and late stages of learning.

### Family factors

A growing body of research has demonstrated that family factors play a very important role in the development of students’ reading skills (Halle et al., 1997; Crosnoe et al., 2010; Hongbo et al., 2016); it even suggests that family engagement is a better predictor of student achievement compared to school engagement (Tatlisu et al., 2011), and that schools cannot compensate for differences in reading skills because of family differences (Banerjee and Lamb, 2016). Studies on family factors influencing students’ reading literacy center mainly on family financial situation and parents’ education level.

#### Family financial situation

It has been found that students’ academic performance is significantly and positively correlated with the family financial situation. In China, Han (2017) found that family income has a significant influence on children’s education level, and the increasing family income can improve their education level. Family influence on reading literacy exists from early childhood. For instance, Crosnoe et al. (2010) claimed that children from families of high socioeconomic status are more likely to be exposed to stimulating environment that are critical to children’s reading, and early learning differences usually exist as the child grows, and the differences in reading skills are more significant across financial levels in the later stages of learning (Welch, 2013; Goldfeld et al., 2021). In addition, Hongbo et al. (2016) showed that families with better financial conditions can provide their children with access to more reading resources and educational chance to promote reading competence, which is consistent with findings of Silin et al. (2014), who indicated that high-ESCS families usually possess more cultural capital. For low-ESCS families, in contrast, there are many mediating variables associated with poor reading development such as higher rates of absenteeism and mobility, and less parental encouragement of academic pursuits (Buckingham et al., 2013).

#### Parents’ educational level

Parental educational level affect student’s ability indirectly through mediating variables such as behaviors (Davis-Kean and Pamela, 2005). From the Greek census data, Davis-Kean and Pamela (2005) found that the educational achievement of daughters in the last 30 years depends significantly on the educational level of parents (especially mothers); Janet and Enrico (2003) also found a significant positive effect of parents’ educational level on children’s educational participation and academic achievement. A number of studies have found that parental education requires some mediating variables to play a role. For example, Mauldin et al. (2001) found that parental education influences the economic investment that families put into their children’s education; this is similar to the conclusion of Spagat (2006), who argued that students with well-educated parents often take advantage of their family background and invest heavily in their human capital. In addition, there is a strong positive relationship between parents’ educational level and time spent on their children (Guryan et al., 2008); moreover, parents’ educational level affects family environment as well as parent-child interaction (Davis-Kean and Pamela, 2005), which in turn indirectly influence children’s academic performance via parental involvement (Xiaoying, 2022).

### School factors

School is the primary place where students live and learn in their daily education. School-level factors that affect students’ reading literacy are mainly school climate and type, teachers’ classroom teaching, among others.

#### School climate

Perparim (2014) stated that school type, school socioeconomic status, and classroom environment were significant predictors of reading performance, as well as differences in the effects of gender and family socioeconomic status on reading performance, and students in poorer schools may not have the material conditions to satisfy their reading achievement demands (Hart et al., 2013). However, Bo et al. (2017) found that school size and material resources had little effect on student performance, but friendly school climate and classroom order could significantly and positively influence student performance. So school climate is also a focus of the research in this regard. Berkowitz (2021) pointed out that schools’ positive social climate could moderate the strength of the association between SES and achievement, and narrow the literacy achievement gap among students under different financial situation. Meanwhile, school climate perceptions can compensate for the negative contribution of disadvantaged family factors on academic achievement (O’Malley et al., 2015).

#### Teacher instruction

As direct guides of students in the learning process, teachers can directly and profoundly influence students’ learning; however, this influence is exercised indirectly through mediating variables that affect students’ academic performance. For instance, teacher’s support can significantly affect students’ academic emotions. Positive emotions were regarded as a mediation variable between teacher support and academic engagement, and teacher’s support could promote positive emotions and mitigate negative ones, resulting in students’ improved academic engagement and enjoyment (Ahmed et al., 2010; Ms and Syh, 2021). Moreover, it has been found that teacher support motivates students to read and thus enhances their reading competence, because students’ interest and attention will grow if they perceive teacher’s support (Law, 2011). In addition, teachers’ perceived school environment (e.g., student support, collegiality, resource adequacy, work pressure) influenced teaching styles (Webster and Fisher, 2003), and teachers’ utilization of appropriate teaching styles, such as providing students with effective and challenging learning tasks and using motivational teaching strategies to stimulate students’ interest in reading, can lead to improved student’s performance in reading comprehension (Law, 2011).

### Research questions

We expand our previous research by exploring a large number of possible student-level predictors gathered during the Program for International Student Assessment (PISA) by utilizing machine learning method to predict reading ability. The machine learning method employed here has notable advantages over traditional statistical analyses, especially for large datasets like PISA, for it is obviously helpful in handling complex interactions among predictors and nonlinear trend, and the regularization methods can help prevent over-fitting of models. The strength of machine learning models have been proved in the study of education tasks such as predicting dropout in MOOCs (Xing, 2019), recommendation-based mobile personalized learning (Hsu et al., 2013) and analyzing student’s emotion and behavior (Ninaus et al., 2019). On the other hand, however, due to a lack of interpretability, researchers have to combine machine learning with interpretation algorithms in exploring the relationship between predictors and outcomes. For example, (Bosch, 2021) used Shapley values to calculate the importance of each variable to mindset interventions, (Hu et al., 2022) utilized Support Vector Machine—Recursive Feature Elimination (SVM-RFE) in classifying the contextual factors influencing reading performance.

In this study, we used the PISA dataset to explore two research questions, which, in addition to having confirmed previous conclusions, reveals some new interesting findings. The questions are:

RQ1: Among individual, family and school factors, which has the greatest impact on reading literacy? Besides providing an answer to this question, we also identify some variables worthy of further discussion.
RQ2: What’s the contribution of each of these variables in predicting reading literacy? In this analysis we have explored whether these variables are monotonic or linear with, and, if not, whether there is an optimal value.

## Materials and methods

### Data source

The data used in this study come from the Program for International Student Assessment (PISA 2018), conducted globally by the Organization for Economic Cooperation and Development (OECD) in 2018, which evaluates whether, at the end of compulsory education, students had the skills and knowledge necessary to participate in the society of the future, rather than focusing only on whether students had mastered specific subjects. PISA 2018 used two-stage stratified sampling to draw the sample. Schools were selected using unequal probability sampling proportional to school size in the first stage, and students were randomly selected from the selected schools to participate in the assessment program in the second stage. In China, a total of 361 secondary schools and 12,058 students from Beijing, Shanghai, Jiangsu and Zhejiang participated in this program.

### Data preprocessing

The 12,058 students (992,302 after weighting) from the above mentioned four provinces/municipalities of China are the objects of our analysis. Since both original questionnaire variables and composite variables were included in the dataset, the above duplicate original questionnaire variables were excluded, some variables that were not measured in the four provinces/municipalities were deleted, and some composite variables were screened, such as ESCS (index of economic, social and cultural status), which was synthesized from highest parental occupation, parental education and home possessions; and learning time, which was obtained by multiplying the weekly class hours by the average length of each class. Nearest-neighbor averaging was applyed by levels to impute missing values of continuous variables, with 134 (10174.7 after weighting) or 1.1% (1.0% after weighting) of the total samples, and we removed samples with missing categorical variables. Finally, a total of 11,924 (982,127 after weighting) samples were retained.

In the PISA data, the cognitive data are scaled with the Rasch Model and the performance of students is denoted with plausible values (PVs) (OECD, 2009a). PISA 2018 provided 10 plausible values (PV values), which are randomly drawn from the student’s trait distribution, to estimate the probability distribution of students’ reading literacy. Statistical analyses usually should be performed independently on each of these 10 plausible values and aggregate results, but, with the large number of samples, using one plausible value or all plausible values does not make any substantial difference (OECD, 2009b). Therefore, the first plausible value (PV1), which was considered as an estimate of students’ reading literacy, was introduced into the statistical model as a predicted variable.

### Model training

Most studies on reading factors used structural equation models (Halle et al., 1997; Sheng et al., 2014; Hongbo et al., 2016; Kaili and Wei, 2021), multilevel regression (Pingping et al., 2011; Bo et al., 2017) and quantile regression (Chunjin, 2020). These methods have many shortcomings. For instance, they are all linear models, which cannot identify the nonlinear relationships and handle complex interaction effect; and they have some strong assumptions such as samples independence and variables independence, which unfortunately are often not correct in reality.

Machine learning methods can provide may inspirational ideas for educational researchers because they require no specific assumption and can explore patterns contained in complex, massive data in a data-driven manner. The application of machine learning in educational task research has become increasingly popular in recent years.

This study used three machine learning models, i.e., Support Vector Regression (SVR), Random Forest Regression (RFR), and eXtreme Gradient Boosting (XGBoost), to predict students’ reading literacy. SVR is a type of linear regression, but a slack variable ∈ is added to the calculation of the loss function, and only sample points that fall outside the interval band of width 2 ∈ are counted. RFR and XGBoost are two classical models of ensemble models, which consist of multiple weak learners. RFR is based on the idea of bagging, generating several regression trees, with each constructed by some randomly selected samples and features. All weak learners learn and make predictions independently, and finally, the predictions of all weak learners are averaged to obtain the final prediction results. XGBoost is a machine learning algorithm proposed by Chen and Guestrin (2016), which has gained wide attention in recent years due to its significant prediction accuracy. XGBoost uses a second-order Taylor series to approximate the value of the loss function and further reduces the possibility of overfitting by adding a regularization term. Besides, overfitting can be further prevented by adjusting parameters such as “maximum tree depth” and “smallest subtree weight” to give the best prediction results in the test set.

10-fold cross-validation was used in this study to adjust the hyperparameters based on the decidable coefficients *R*^2^ on the test set, and finally the combination of hyperparameters that performed best on the test set was selected, with the model showing highest predictive ability being chosen.

### Model interpretation

The complexity of the models of machine learning makes it hard to provide interpretability despite their improving prediction accuracy. That means that we are unable to explain how the models use features and samples to make decisions (Molnar, 2020). The lack of interpretability has discouraged the application of machine learning methods to some extent. Therefore, many scholars in applied research still prefer to use simple models that are easy to interpret. To tackle this problem, some interpretation algorithms have been proposed, including linear regression and logit regression. For example, Local Interpretable Model-Agnostic Explanations (LIME) (Ribeiro et al., 2016) and SHapley Additive exPlanations (SHAP) (Lundberg et al., 2018) are usually used to interpret individual predictions; Partial Dependence Plot (Friedman, 2001) and Accumulated Local Effects Plot (Apley and Jingyu, 2016) are used to describe trends between variables and outcomes. Some studies used simple interpretable models (e.g., decision trees) to fit complex models and results in providing interpretation for the models (Wan et al., 2020; Wu et al., 2020). This study used the SHAP method to provide an explanation of the trained model and carried out data-driven analysis to explore factors influencing reading literacy from a global perspective.

The Shapley value is a method from coalitional game theory that can be used in machine learning to measure the contribution of each variable when the model makes predictions for a particular instance, where contribution refers to the difference between the impact of a variable and the average impact. The Shapley value for each feature value is obtained by weighting and summing the marginal contributions of all possible combinations of feature values.


(1) $$\phi_{j}=\sum_{S\subseteq \left\{x_{1},x_{2},\cdots ,x_{p}\right\}\backslash \left\{x_{j}\right\}}\frac{\left|S\right|!\,\left(p-\left|S\right|-1\right)!}{p!}\,\left(v\,a\,l\,\left(S\,⋃x_{j}\right)-v\,a\,l\,\left(S\right)\right)$$

where *S* is a subset of the features used in the model and *val*(*S*) denotes the result of the prediction using the subset *S*.

When there are more features, the traversal of all combinations of features makes the computational process too complex. To address this problem, Lundberg et al. (2018) developed a fast and accurate algorithm for tree models, which reduces the computational complexity by computing all possible subsets in parallel while traversing all nodes along the decision path using the structural properties of the tree model. SHAP values, with their properties of local accuracy, missingness and consistency, can perform both local and global interpretability and are an effective method for explaining various machine learning models. SHAP values interpret the predicted values of the original model approximately as the sum of effects of all feature attributions.


(2) $$f\left(z^{\prime }\right)=\phi_{0}+\sum_{i=1}^{p}\phi_{i}{z^{\prime }}_{i}$$

where ϕ_0_ is the predicted mean of all training samples. *z*′ ∈ {0,1}^*p*^, where p is the number of features. z′i typically represents a feature being observed (z′i=1) or unknown (z′i=0), and ϕ_*i*_’s are the effects of feature attributions. SHAP values are calculated separately for each variable of each sample, and the global contribution of each variable is obtained by taking the absolute average of the SHAP values of all samples of the same variable.


(3) $$\phi_{j}=\frac{1}{N}\sum_{i=1}^{N}\left|\phi_{ij}\right|,j=1,2,\cdots ,p$$

where N is the number of samples.

## Results

### Descriptive statistics

The python 3.8 programs were used in the study. The results of descriptive statistics and correlation coefficient with PV1 (*M* = 557.15, *SD* = 92.58) were shown in Table 1. It was found that at the individual student level, the three reading metacognitions (understanding and remembering, summarizing, and assessing credibility) and reading interest were the most strongly correlated with PV1; at family level, ESCS and family wealth were the most correlated with PV1; features at school level were less correlated with PV1 compared to the other two, and it was total learning time, discipline climate in the subject, and teachers’ stimulation of students to engage in reading that show the highest correlations with PV1.

**TABLE 1:** Descriptive statistics for continuous variables.

Variables			minimum	maximum	Mean	Std	r	Variables			minimum	maximum	Mean	Std	r
Individual variables	Meta-cognition	Understanding and remembering	–1.640	1.500	0.170	0.996	0.313***	Family Variables	Household possessions	Cultural possessions at home	–2.613	2.053	–0.218	1.105	0.253***
		Summarizing	–1.720	1.360	–0.114	0.956	0.347***			Home educational resources	–4.411	1.210	0.149	1.027	0.231***
		Assess credibility	–1.410	1.330	0.049	0.961	0.452***			ICT resources	–3.768	3.601	–0.579	0.881	0.249***
	Expected occupational status		0.000	88.960	55.528	29.812	0.239***			Family wealth	–6.984	4.225	–0.814	0.834	0.296***
	Reading related attitudes	Reading interest	–2.711	2.657	0.971	0.855	0.303***	School variables	Test language lessons	Disciplinary Climate	–2.712	2.035	0.793	1.029	0.174***
		Perception of competence	–2.440	1.884	0.018	0.855	0.174***			Teacher support	–2.711	1.341	0.365	0.888	0.055***
		Perception of difficulty	–1.89	2.78	0.116	0.951	–0.236***			Teacher-directed instruction	–2.943	1.820	0.486	1.027	–0.052***
	Dispositional variables	Learning activities	–2.538	1.084	0.131	0.928	0.135***			Perceived feedback	–1.639	2.017	0.279	1.032	0.010***
		Competitiveness	–2.345	2.005	0.409	0.810	0.125***			Teacher’s stimulation of reading engagement	–2.300	2.087	0.560	1.030	0.170***
		Work mastery	–2.737	1.816	0.277	0.888	0.104***			Adaptation of instruction	–2.265	2.007	0.376	1.036	0.094***
		General fear of failure	–1.894	1.891	0.002	0.863	0.035***			Perceived teacher’s interest	–2.218	1.825	0.293	0.968	0.129***
		Resilience	–3.168	2.369	–0.120	0.938	0.035***		School Climate	Perception of competitiveness	–1.989	2.038	0.176	0.913	0.075***
		Mastery goal orientation	–2.525	1.852	–0.011	0.904	0.143***			Perception of cooperation	–2.143	1.676	0.184	1.001	0.057***
	Students’ well-being	Eudaemonia: meaning in life	–2.146	1.741	0.079	0.901	–0.104***			Sense of belonging to school	–3.258	2.756	–0.188	0.873	–0.054***
		Positive affect	–3.067	1.239	0.111	0.884	0.018***			Experience of being bullied	–0.782	3.859	–0.200	0.883	0.075***
Family Variables	ESCS		–5.077	3.102	–0.662	1.067	0.368***		Learning time	Learning Time (Reading)	0.000	2400.000	283.650	141.363	–0.075***
	Duration in early childhood education and care		0.000	7.000	3.118	0.840	0.070***			Total Learning Time	150.000	3000.000	1816.160	503.588	0.178***
	Parents’ emotional support		–2.447	1.035	–0.021	0.916	0.171***								

***p < 0.01.

### Comparison of models

Three models were trained with 59 variables filtered to predict reading literacy PV1 values. To avoid overfitting, the hyperparameters of the three models mentioned above were optimized separately. A 10-fold cross-validation was performed in adjusting the parameters to select the optimal hyperparameter combinations on the test set. Table 2 shows the optimal hyperparameter combinations for the above three models, where the hyperparameter C of SVR is the relaxation variable penalty coefficient, gamma is inversely proportional to the standard deviation of the radial basis kernel function. RFR and XGBoost has more hyperparameters and using the grid search method would result in too much computation, so each hyperparameter has been optimized in turn in the order shown in Table 2. The optimized predictive abilities of the three models are shown in Table 3. The predictive ability were measured by the *R*^2^, Root Mean Square Error (RMSE) and Pearson’s correlation r. All coefficients were calculated after weighting and explained in detail as follows.


(4) $$R^{2}=1-\frac{\sum_{i}w_{i}\,{\left({\hat{y}}_{i}-y_{i}\right)}^{2}}{\sum_{i}w_{i}\,{\left(y_{i}-\bar{y}\right)}^{2}}$$

(5) $$R\,M\,S\,E=\sqrt{\frac{1}{\sum_{i}w_{i}}\,\sum_{i}w_{i}\,{\left({\hat{y}}_{i}-y_{i}\right)}^{2}}$$

(6) $$r=\frac{\sum_{i}w_{i}\,\left(x_{i}-\bar{x}\right)\,\left(y_{i}-\bar{y}\right)}{\sqrt{\sum_{i}w_{i}\,{\left({\hat{x}}_{i}-x_{i}\right)}^{2}}\,\sqrt{\sum_{i}w_{i}\,{\left({\hat{y}}_{i}-y_{i}\right)}^{2}}}$$

**TABLE 2:** Optimal hyperparameter combination of the three models.

Models	Hyperparameter combinations	Optimal hyperparameter combination
SVR	C=(0.1,1,10,100) gamma=(0.0001,0.001,0.01,0.1,1)	C=1 gamma=0.001
RFR	n_estimators=(500,2000,by=100) max_depth=(20,50,by=5) min_samples_leaf=(1,2,3,4,5,6) min_samples_split=(1,2,3,4) max_features=(10,30,by=1)	n_estimators=1000 max_depth=22 min_samples_leaf=3 min_samples_split=3 max_features=24
XGBoost	n_estimators=(500,2000,by=100) max_depth=(15,30,by=1) min_child_weight=(1,2,3,4,5) gamma=(0.1,0.2,0.3,0.4,0.5) subsample=(0.5,0.6,0.7,0.8,0.9) colsample_bytree=(0.5,0.6,0.7,0.8,0.9) reg_alpha=(0,0.1,1,2,3) reg_lambda=(0,0.1,1,2,3) learning_rate=(0.01,0.1,1,2,3)	n_estimators=1800 max_depth=20 min_child_weight=3 gamma=0.2 subsample=0.7 colsample_bytree=0.8 reg_alpha=0.1 reg_lambda=0 learning_rate=1

**TABLE 3:** Comparison of the ability to predict and fit of the three models.

	5-fold cross-test set prediction effect		
	
	R^2^	RMSE	Pearson’s *r*
SVR	0.4660	63.4415	0.6854
RFR	0.4732	63.1013	0.6918
XGBoost	**0.4802**	**62.6765**	**0.6965**

After 10-fold cross-validation, the prediction results of the three models were found to be similar, and XGBoost performed slightly better than the remaining two models. Therefore, the XGBoost model was chosen to fit all the data in this study, which was interpreted by the SHAP values.

### Feature analysis

**FIGURE 1:**
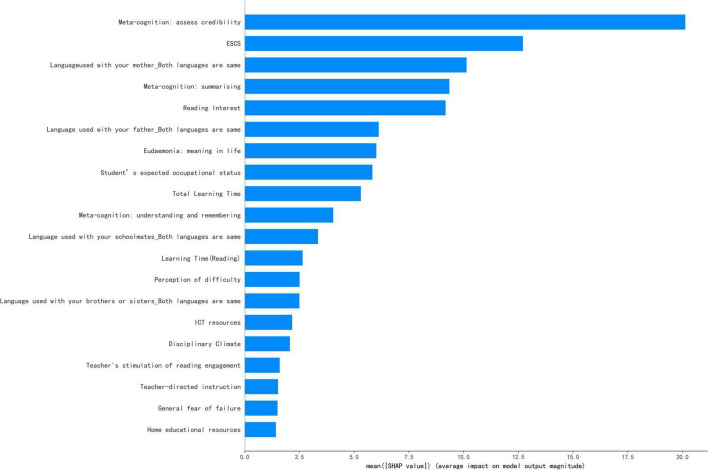
The average absolute SHAP value indicates the feature contribution.

**FIGURE 2:**
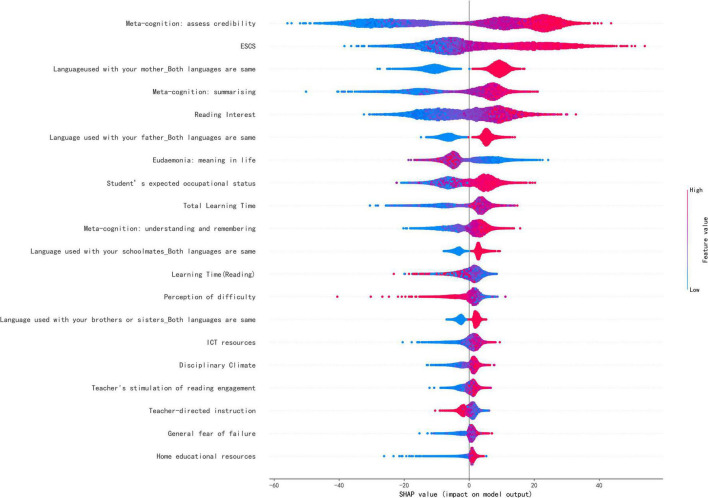
SHAP summary plot.

The SHAP values can calculate the contribution of each feature to each sample. Because features have both positive and negative effects on the sample, to measure the global importance of certain features, we take the absolute average of the SHAP values of the feature for all samples. The global contribution values of each variable are shown in Supplementary Appendix 1. Figure 1 shows the top 20 features with the highest global contribution values. Consistent with the results of the descriptive statistics, the features at individual student level and at family level rank higher than those at school level, and the highest contributing feature at school level, “total learning times,” ranks only 10th. At individual student level, three reading metacognitive strategies were important predictors of reading literacy, in addition to reading interest, wellbeing, and expected occupational status; at family level, ESCS and the language used to communicate with family members were the most contributing features; and at school level, learning time and discipline in the classroom were the most important features. The SHAP summary plot was drawn to more clearly visualize the directionality of each feature’s influence on reading literacy, as shown in Figure 2.

Figure 2 shows the feature importance and direction of the effect of each feature on reading literacy. Each point in the figure is the SHAP value of a feature of an instance, with the position on the vertical axis indicating the feature value and the position on the horizontal axis indicating the SHAP value, and the feature values of all instances plotted with different color dots, where red dots and blue dots represents high feature values and low feature values, respectively. Taking the first feature, “metacognition: assess credibility” as an example, the SHAP value corresponding to the blue dot is negative, which means that the low feature value decreases reading literacy from the average of all samples, and the SHAP value corresponding to the red dot is positive, which means that the high feature value increases reading literacy, so the feature has a positive effect on reading literacy.

**FIGURE 3:**
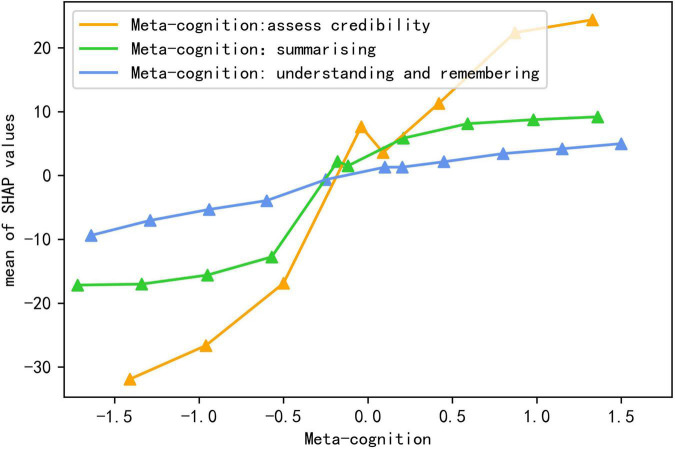
The line chart of meta-cognitions and Shap values.

This study explored the most important predictors at the levels of the individual student, family, and school. First, at individual level, the strongest predictors of reading literacy were reading metacognition and reading interest. The global contributions of the three reading metacognitions were 20.127, 9.350, and 4.045, respectively, and the line chart of feature values and SHAP values were created to better visualize the effect of metacognitions on reading literacy (see Figure 3). As shown in Figure 3, all three reading metacognitions showed a positive relationship with reading literacy, which is corresponding with the transition from blue to red in the Figure 2. Therefore, reading metacognition had a positive predictive effect on reading literacy.

**FIGURE 4:**
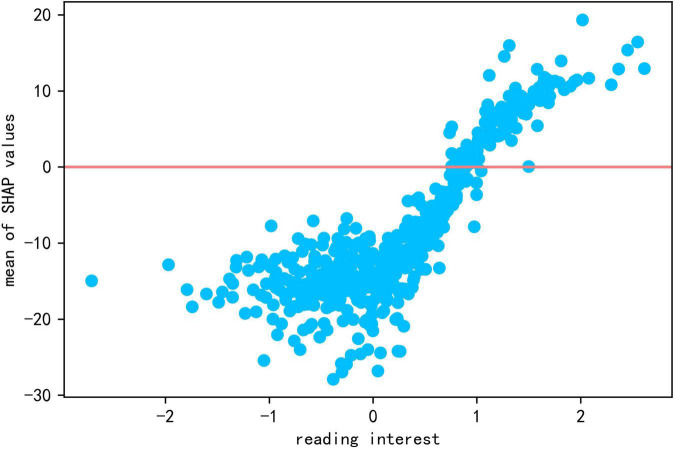
The scatter plot of reading interest and Shap values.

Reading interest had a strong influence on students’ reading literacy, with a global contribution value of 9.176. As seen in Figure 2, the blue points of reading interest (low reading interest) correspond to negative SHAP values, and the red points (high reading interest) to positive SHAP values. SHAP values increase with the growth of feature values. The scatter plot of interest and SHAP values (see Figure 4) also shows a positive relationship with reading literacy, with students who are more interested in reading being more likely to perform at a higher level of reading literacy.

**FIGURE 5:**
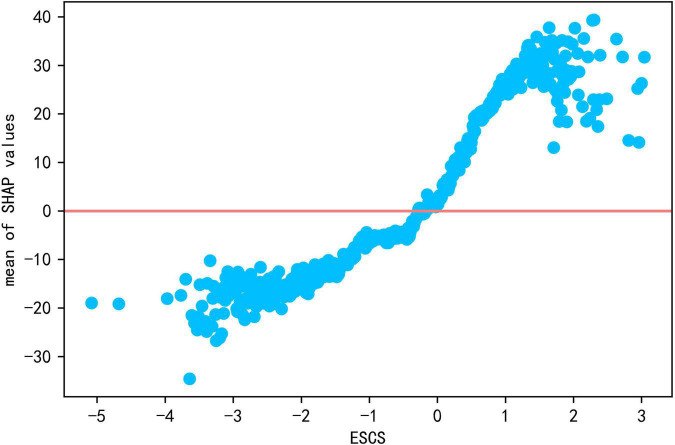
The scatter plot of ESCS and Shap values.

The strongest predictors at family level are ESCS and home language environment. The global contribution value of ESCS is 12.708, the second strongest predictive feature after “metacognition: assess credibility.” As seen in Figure 2, the color of this feature gradually transitions from blue to red along the positive horizontal axis, i.e., low ESCS corresponds to negative SHAP values, and high ESCS to positive ones. It can also be seen from the scatter plot of ESCS and SHAP values (see Figure 5) that there is a significant positive relationship between ESCS and reading literacy.

The categorical variable, which is one-hot coded in preprocessing, is the language used in communication with the mother and father, siblings, and classmates. The higher SHAP value is for the 0−1 variable generated from the fourth option in the questionnaire – “heritage language and test language are the same,” i.e., whether there is a dialect. The global contribution values for “heritage language and test language are the same” when communicating with mother, father, siblings, and classmates are 10.133, 6.124, 2.499, and 3.349, respectively. Because the homogeneity of variance was rejected by our data, Kruskal-Wallis test was used in the comparison among multiple groups. Table 4 shows the result of Kruskal-Wallis test for language used with mothers, fathers, siblings, and classmates, and reveals that the rank mean of category 4th is significantly higher than those of the other three categories. In addition, although the global contribution values for category 2 (“About equally often my heritage language and test language”) are small, at 0.094, 0.075, 0.069, and 0.013, the results of test shows that the mean rank for category 2th is significantly higher than the two remaining categories for language of communication with family members (“mostly my heritage language” and “mostly test language”). In summary, students without dialect have significantly higher reading literacy than others, while among the latter, students who use a dialect with the same frequency as Mandarin with family members have significantly higher reading literacy than those who use 0 only one of the languages.

**TABLE 4:** Result of Kruskal-Wallis test and rank mean for all groups for language used with family members and classmates.

Which language do you usually speak with	Mostly my heritage language	About equally often my heritage language and test language	Mostly test language	Heritage language and test language are the same	*χ* ^2^
Mother	362326.80	445913.78	376516.80	585630.66	130125.459***
Father	364719.22	442957.02	364719.22	584581.07	128144.392***
Brothers and sisters	342983.29	431139.04	394664.92	583486.07	128416.819***
Classmates	335235.40	339797.87	405090.71	585740.23	129773.874***

***p < 0.01.

School-level features generally lag behind individual level and home level features in the contribution ranking, with the highest contributions coming from learning time and discipline in the classroom. The global contribution values for total learning time and learning time (reading) are 5.306 and 2.646, respectively.

**FIGURE 6:**
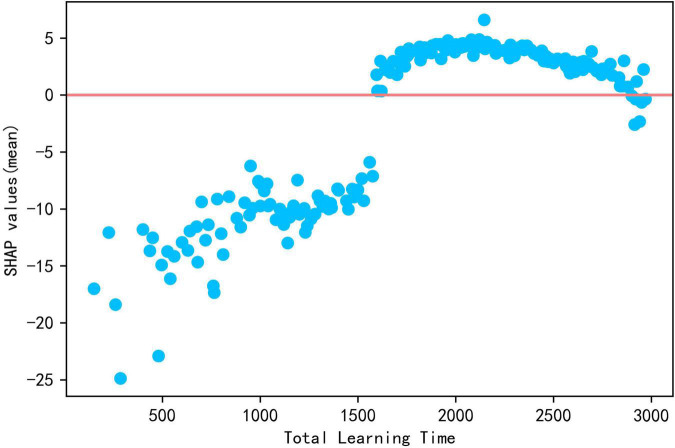
The scatter plot of total learning time and Shap values.

As seen in Figure 2, the blue points for total learning hours correspond to negative SHAP values, indicating that less learning time reduces reading literacy, but the purple and red points overlap each other in the graph, indicating that the SHAP values for moderate learning time are approximately equal to those for high learning time. Drawing the scatter plot shown in Figure 6, the relationship between total learning time and reading literacy is not simply positive and linear, indicating that SHAP values are less than zero, explaining why reading literacy of those sample are less than average, before 1,600 mins, then promote reading literacy from average after more than 1,600 mins. However, SHAP values first increase and then decrease until approximately 3,000 mins in which they are less than zero again. The results above indicates that prolonging time dedicated to learning to more than 1,600 mins makes no further contribution to improving reading literacy.

**FIGURE 7:**
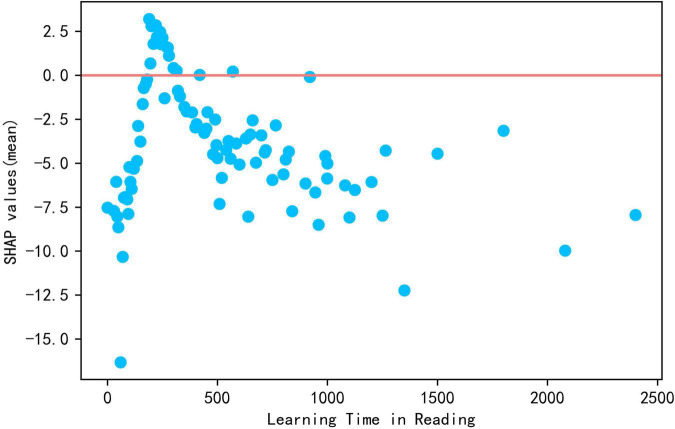
The scatter plot of reading learning time and Shap values.

From the descriptive statistics, it was found that learning time (reading) shows a significant negative correlation with reading literacy at −0.075. From Figure 2, it is known that SHAP values less than zero appear in red and blue at the same time, indicating that lower or higher learning time in reading reduces reading literacy. Plotting the scatter plot of this variable and the SHAP value in Figure 7, its SHAP value has a clear peak at approximately 200–300 mins (approximately 6 h or so) per week when the SHAP value is greater than zero, but as reading time increases, the SHAP value begins to decline and keeps being greater than zero until 300 mins per week. Then the SHAP value less than zero has a negative effect on reading literacy. Therefore, there is an optimal length of total learning time and learning time in reading, which should be controlled within an appropriate range to avoid putting too much burden on students; otherwise, it would be detrimental to students’ academic performance.

**FIGURE 8:**
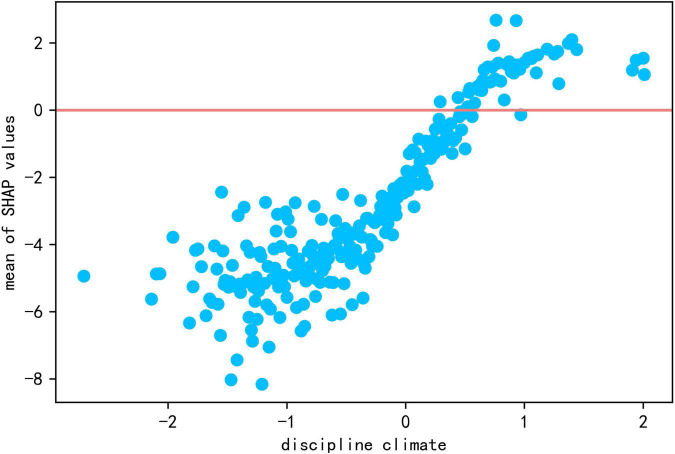
The scatter plot of discipline climate and Shap values.

The global contribution of discipline climate is 2.064. As seen in Figure 2, the blue points of discipline climate (more chaotic classroom climate) corresponds to negative SHAP values, and the red points (better classroom climate) to positive SHAP values. As seen from the scatter plot of discipline climate and it’s SHAP values in Figure 8, discipline climate shows a positive relationship with reading literacy, indicating that it is more likely for students in orderly classroom to demonstrate higher reading literacy.

## Discussion

Based on PISA 2018 dataset from four provinces/municipalities of China—Beijing, Shanghai, Jiangsu, and Zhejiang—this study used the XGBoost machine learning method to construct a prediction model and SHAP values to calculate the contribution of each feature to effectively predict reading literacy and interpret how it was influenced. Next, the influences that contribute the most to reading literacy were discussed at individual level, family level, and school level, respectively.

### Individual level

**Conclusion** 1: Reading metacognition has a strong influence on reading literacy, with assessing credibility being the strongest predictor, summarizing the second, and understanding and remembering the weakest.

Based on the global contribution values calculated for each variable, reading metacognition was found to be an effective predictor, especially assessing credibility and summarizing. Reading metacognition refers to the extent to which the individual knows effective learning strategies (Chunjin, 2020). Learning strategies can be divided into deep learning strategies, which refer to attempts to integrate new information with prior knowledge, and surface learning strategies, which involve repetitive rehearsal and rote memorization of information (Murayama et al., 2013). Assessing credibility and summarizing can be categorized as deep learning strategies and understanding and remembering be regarded as surface learning strategies. The results indicates that the perception of effectiveness of deep learning strategies is far more important in the model than the perception of effectiveness of surface learning strategies because deep learning strategies promote connections between concepts and understanding of learning material; in contrast, surface learning strategies does not help students integrate new information into existing bodies of knowledge (Murayama et al., 2013). Some studies (Areepattamannil and Caleon, 2013; Murayama et al., 2013) suggested that remembering strategies shows an inverse relationship with mathematical literacy, but both Chunjin (2020) and the present study have found a positive predictive effect of remembering strategies on reading literacy, which may be due to the different disciplinary characteristics between reading and mathematics. Students’ ability to use strategies could regulate the level of language learning through cognitive strategies (Ghafournia and Afghari, 2013), and the use of effective learning strategies also promotes students’ engagement in learning (Lee and Shute, 2010); therefore, improving students’ ability to use cognitive strategies is crucial to improving reading proficiency. Paris and Oka (1986) argued that teachers can provide metacognitive knowledge about effective strategies to improve students’ reading skills, and Qishan et al. (2018) found that teacher’s instruction can influence students’ reading literacy not only directly but also indirectly through students’ learning strategies and reading metacognitive strategies. Therefore, teachers should realize that rote memorization does not improve students’ reading ability efficiently. What teachers should do is not only to lead students to read but also instruct them how to read, and the most effective way to improve students’ reading literacy is to help them master reading strategies and metacognitive strategies through teacher guidance.

**Conclusion** 2: Interest in reading can have a positive impact on reading literacy.

Reading interest is a significant positive predictor of reading literacy, which is consistent with the findings of many studies (Wenjing and Tao, 2012; Lechner et al., 2019; Chunjin, 2020). In exploring how reading interest affects reading literacy, Schraw et al. (1995) argued that students with greater interest in a text always pay more attention to reading that text. Hongbo et al. (2016) argued that reading interest indirectly affects reading literacy by influencing reading engagement and that students who spend more time reading because of their interest are more likely to be good readers. If students are interested in reading, they are more likely to spend more attention, energy and time on learning and reading and, thus, gain more knowledge. It has also been found that the text itself also affects reading interest, and the relevance, vividness, and comprehensibility of the text are closely related to reading interest (Schraw and Dennison, 1994). Schraw and Dennison (1994) found that the level of interest in a text is related to the student’s perspective and that a specific reading purpose increased the level of interest in that text; the more vivid and comprehensible the text is, the more interest of students will be aroused, and the higher the level of text recall, the more the knowledge gained. In addition, a number of studies have found that teacher support can influence reading engagement and reading interest and indirectly affect students’ reading literacy (Kaili and Wei, 2021). Therefore, teachers should provide appropriate support from various aspects, such as emotional care, learning instruction, ability guidance, in addition to focusing on cultivating students’ reading interests in reading teaching practice, taking the vividness of reading materials into consideration when selecting reading texts, giving instruction in conjunction with students’ interests, and helping students understand the relevance of textual knowledge in an interest-oriented manner to increase their reading level.

### Family level

**Conclusion 1**: High family ESCS is advantageous for reading literacy.

This study found that the ESCS is a strong predictor on reading literacy, and the higher the ESCS, the more beneficial it was for reading literacy. Welch (2013), Silin et al. (2014) also pointed out that there is a significant relationship between family financial level and academic performance, with children from families with high ESCS having an advantage over those with low ESCS. Tatlisu et al. (2011), Banerjee and Lamb (2016) even argued that family background has a much greater impact on students than school influence, further illustrating the importance of family factors. Low-ESCS families usually have less time, resources, or means to engage in their children’s education, and parents place less emphasis on books, reading, and education (Banerjee and Lamb, 2016). In contrast, high-ESCS families usually possess more cultural capital and a greater ability to create a good learning environment (Silin et al., 2014), so that their children usually have more opportunities to devote themselves to reading and learning. Even when their children do not do well in school, high-ESCS families are more able to afford high tuition and provide extracurricular learning resources to assist their children’s learning (Mauldin et al., 2001). Families with high ESCS have more educational advantages than families with low ESCS, and over time, advantaged families would occupy more quality educational resources and opportunities, and disadvantaged families would be increasingly disadvantaged (Silin et al., 2014), which goes against the principle of educational equity. Although studies have shown that school interventions cannot completely eliminate the gap caused by family financial situations (Goldfeld et al., 2021), student reading strategies, interest in reading, school operations, and teacher-student relationships have been proved to have a significant contribution to the academic performance of low ESCS students (Hao and Yifang, 2020; Mingman and Guomin, 2021).

**Conclusion** 2: Home language environment has an impact on students’ reading literacy, with dialects having a negative effect on reading literacy; however, proficiency in both a dialect and Mandarin has a positive effect on reading literacy.

The results indicate that home language environment has a strong influence on students’ reading literacy. After one-hot coding of the language variables used, it is found that when the “heritage language and test language are the same” (i.e., whether there is a dialect), there was a greater impact of the home language environment on reading literacy. This study found that dialects negatively influence students’ reading literacy. Due to the difference between spoken or written dialects and the official lingua franca, students whose native language is a dialect often make grammatical mistakes and showed poor expressions when learning Mandarin, which can also hinder their written reading and writing (Qin and Zhe, 2020; Yinyin, 2020). Some studies have shown that children with a Mandarin dialect perform worse in the concepts of word (awareness of words as separate units) and syntactic awareness (syntactic knowledge and competence) than Mandarin monolingual children (Hanling and Rongbao, 2014; Yongxiang and Rongbao, 2015). However, dialects are not always harmful, and Hanling and Rongbao (2014) noted that dialect experience has a positive effect on the development of children’s sense of denotational arbitrariness (the extent to which children understand the conventional relationship between language as a symbol and the meaning it refers to) due to bilingual children’s greater metalinguistic awareness, i.e., their ability to distinguish between two language systems early in language learning and to suppress non-target language during language use (Wenyu, 2010), which allows them to perform better than monolingual children on all metalinguistic tasks requiring a high degree of controlled processing and to generalize the advantage of controlled attention to other nonlinguistic domains (Bialystok, 1988). In addition, bilingual children had different language learning strategies and could learn new languages faster than monolingual learners (Nayak et al., 1990). Bialystok (1988) suggested that these advantages of bilingual children are contingent on a good understanding of the second language and becoming “balanced” bilinguals, which explained why the negative effect of dialect often plays out in the early years of language learning, and the disadvantage of dialect-common speaking children disappear when children moved into higher grades (Sumei et al., 2013). This was also supported by our study, which found that students in all three categories, except “heritage language and test language are the same,” using both dialect and Mandarin with family members rather than classmates have significantly higher reading performance than students who use only one language because the former are closer to the “balanced” bilinguals. In China, students usually are required to speak *Mandarin* in school, so we suggest parents, in dialect areas, to not ignore teaching of dialects and provide children with opportunities to practice dialect at home so that kids could become “balanced” bilinguals and give full play to the lingual advantages of bilingual children.

### School level

**Conclusion** 1: Learning time positively predicted reading literacy, but longer learning time does not improve academic performance and may even be the reverse.

Some studies (Yan and Leung, 2012; Kidron and Lindsay, 2014; Jez and Wassmer, 2015) indicated that longer learning times improves students’ academic performance, and the present study gets a similar conclusion. However, the difference is that the present study found that increasing learning time is of limited help to improving achievement, with optimal total learning time for improving academic performance being about 1,600 mins or 26.6 h per week and learning time in reading 200 to 300 mins or 6 h per week. Exceeding this time limits would may be detrimental to academic performance instead of improving it. This is consistent with the findings of Xuejun (2014), Fei and Hongbo (2017) that, although learning time is positively correlated with academic performance, too much time spent on learning is not necessarily better. Bloom (1974) distinguished between “elapsed time” (time presumably working on the task) and the actual time spent on the task, with the latter closely related to learning achievement. Extending learning time does not necessarily increase the time spent on work but rather increased students’ coursework burden (Linchun and Lujian, 2007; Mengjie and Tao, 2019), which leads to negative emotions such as anxiety and aversion to learning (Xiao, 2013), which may lead students to flee and avoid learning, resulting in negative academic performance (Arsenio and Loria, 2014). Our study suggests that teachers and parents should realize that reducing students’ play and rest time to unnecessarily extend learning time does not do much for improving academic performance. Chinese officials have begun to call for reducing the burden of homework and out-of-school training for students in compulsory education, and teachers should reasonably control class time and learning time to effectively help students improve their academic performance.

**Conclusion 2:** Maintaining discipline climate was useful for improving reading literacy.

This study has found that discipline climate was a significant positive predictor of reading literacy, which was in line with the findings of Arens et al. (2015), Simba et al. (2016) and others. Discipline played a very important role in students’ academic performance; students with a sense of discipline are more focused, study harder, show greater determination and are more likely to be accepted and appreciated by teachers and parents, thus develop more positive self-identity and have more motivation (Simba et al., 2016), while disruptions in classroom order could be detrimental to teaching efficiency and may even interrupt normal teaching and learning activities. Some studies have shown that teachers’ content, delivery process and teaching attitude are important factors in students’ classroom disruptions (Guiping et al., 2005), and that most classroom disrupters are students who are not engaged in learning, are bored and, as a result, would miss learning opportunities; furthermore, their dissatisfaction with learning exacerbated disengagement (Rahimi and Karkami, 2015; Lopes and Oliveira, 2017). Therefore, in addition to acquiring necessary academic and pedagogical skills, teachers need to acquire management skills to ensure order in the class (Lopes and Oliveira, 2017). While legitimate use of punishment helps to maintain the authority of discipline and could have the effect of maintaining discipline in the short term, overreliance on punishment strategies cannot necessarily ensure long-term stability in class order (Mark, 2006) and may even exacerbate classroom disruptions and stimulate student resistance and hostility (Roache and Lewis, 2011). Sun (2015) held that setting transparent rules, talking after class, using punishment strategies appropriately, building positive relationships with students who were mutually trusted, and promoting student engagement in learning were effective discipline strategies. Maintaining order in class was a major concern for most new teachers (Sutton and Wheatley, 2003); thus, not only instruction in teaching competencies but also effective discipline strategies should be included in preservice training for teachers to maintain order in the classroom and ensure that instructional activities could be carried out properly.

## Conclusion and limitations

Based on data from four provinces/municipalities of China in PISA 2018, this study explored the most effective predictors for reading literacy using the XGBoost model and the SHAP values and found that individual-level and family level features had a more significant impact on reading literacy. Reading metacognition at the individual level was the strongest predictor of all variables, in addition to reading interest as an effective positive influence, indicating that guiding students to master appropriate reading strategies and stimulating reading interest are useful for improving students’ reading literacy. Family level ESCS and family language environment are effective predictors of reading literacy: ESCS has a strong influence and positively predicted reading literacy; speaking a dialect was detrimental for reading literacy, although “balanced bilinguals” who were proficient in both a dialect and Mandarin had an advantage over monolingual students. At school level, there was an optimal value for total learning time and reading learning time. Extending learning time cannot improve academic performance, may play a negative role instead; thus, parents and teachers should reasonably control class time and learning time. In addition, discipline climate is beneficial for students’ reading literacy.

There were several limitations of this study that need to be noted. First, because the data in this study are cross-sectional, it is difficult to determine whether there is a causal relationship between the variables, and the variable contributions derived from this study are only predictive contributions rather than causal analyses. The variable attributions through SHAP values make it possible to simply examine a variable in isolation, but in fact, these variables have complex interactions and causal networks. For instance, school and family factors not only have an impact on reading literacy, but also affect individual-level factors. Therefore, it is recommended that future research focus on the mechanisms of influence between variables. Second, while this study obtained some valuable and interesting conclusions using XGBoost, there may be a risk of overfitting using machine learning as the hierarchical data contains multiple levels. Therefore, considering the hierarchical characteristics of PISA data, Future analysis may consider combining machine learning with a mixed effects model to further validate the conclusions of our study. Third, the data used in this study was made up of four provinces/municipalities of China, which were the most developed regions in China and was not representative of students across the country. Forth, considering that among the four provinces/municipalities of China, Shanghai, Jiangsu, and Zhejiang were dialect regions, the high effect of language variables in this study may actually be attributed to regional differences.

## Data availability statement

Publicly available datasets were analyzed in this study. This data can be found here: https://www.oecd.org/pisa/data/2018database/.

## Author contributions

HL provided research guidance and wrote the manuscript. XC analyzed the data and wrote the data analysis reports. XL polished and revised the manuscript. All authors contributed to the article and approved the submitted version.
