# Supplementary material for: Factors influencing secondary school students’ reading literacy: An analysis based on XGBoost and SHAP methods
Source: Front Psychol. 2022 Sep 23;13:948612. doi: 10.3389/fpsyg.2022.948612 (PMC9541006; doi:10.3389/fpsyg.2022.948612)
Supplement: Supplementary file 1 [file Table_1.docx]

**Appendix 1**

Table of global contribution values for all variables.

| Variables | Global SHAP | Variables | Global SHAP |
| --- | --- | --- | --- |
| Meta-cognition: assess credibility | 20.127 | Work mastery | 0.739 |
| ESCS | 12.708 | Perceived teacher's interest | 0.679 |
| Languageused with your mother_Both languages are same | 10.133 | Teacher support | 0.671 |
| Meta-cognition: summarising | 9.350 | Perception of competence | 0.647 |
| Reading Interest | 9.176 | Resilience | 0.645 |
| Language used with your father_Both languages are same | 6.124 | Subjective well-being: Positive affect | 0.565 |
| Eudaemonia: meaning in life | 6.013 | Attitude towards learning activities | 0.546 |
| Student’s expected occupational status | 5.833 | Perception of cooperation | 0.469 |
| Total Learning Time | 5.306 | Duration in early childhood education and care | 0.359 |
| Meta-cognition: understanding and remembering | 4.045 | Gender_F | 0.309 |
| Language used with your schoolmates_Both languages are same | 3.349 | Experience of being bullied | 0.288 |
| Learning Time(Reading) | 2.646 | Language used with your friends_Mostly test language | 0.198 |
| Perception of difficulty | 2.508 | Language used with your schoolmates_Mostly test language | 0.164 |
| Language used with your brothers or sisters_Both languages are same | 2.499 | Language used with your friends_Heritage language | 0.143 |
| ICT resources | 2.165 | Languageused with your mother_Mostly test language | 0.132 |
| Disciplinary Climate | 2.064 | Gender_M | 0.128 |
| Teacher's stimulation of reading engagement | 1.599 | Language used with your brothers or sisters_Heritage language | 0.128 |
| Teacher-directed instruction | 1.523 | Language used with your schoolmates_Heritage language | 0.127 |
| General fear of failure | 1.503 | Languageused with your mother_Heritage language | 0.122 |
| Home educational resources | 1.428 | Languageused with your mother_Equal frequency of use in both languages | 0.094 |
| Mastery goal orientation | 1.425 | Language used with your brothers or sisters_Mostly test language | 0.075 |
| Family wealth | 1.413 | Language used with your father_Equal frequency of use in both languages | 0.075 |
| Parents' emotional support | 1.280 | Language used with your brothers or sisters_Equal frequency of use in both languages | 0.069 |
| Cultural possessions at home | 1.109 | Language used with your father_Mostly test language | 0.066 |
| Perception of competitiveness | 1.024 | Language used with your father_Heritage language | 0.058 |
| Language used with your friends_Both languages are same | 0.985 | Language used with your schoolmates_Equal frequency of use in both languages | 0.013 |
| Competitiveness | 0.833 | Language used with your friends_Equal frequency of use in both languages | 0.012 |
| Adaptation of instruction | 0.829 | Language used at home most of the time_Mostly test language | 0.007 |
| Sense of belonging to school | 0.812 | Language used at home most of the time_other | 0.003 |
| Perceived feedback | 0.746 |  |  |
